# Supplementary material for: De Novo Transcriptome Profiling of Naegleria fowleri Trophozoites and Cysts via RNA Sequencing
Source: Pathogens. 2023 Jan 22;12(2):174. doi: 10.3390/pathogens12020174 (PMC9959186; doi:10.3390/pathogens12020174)
Supplement: Supplementary file 1 [file pathogens-12-00174-s001.zip › Supplementary Table 1 (Table S1).pdf]

Supplementary Table 1. Sequence of primers used for cloning of *Nf-profilin* gene

Name	Primer sequence
PF-1	5’ ATG TGC TTG GAC TCC TTT GCG 3’
PF-3	5’ ATG GCA TCA TCC AAA AGG GT 3’
PR-4	5’ ACC AGC CAA ATT CTT AGC TT 3’
PR-5	5’ GGA AGT TTT CTT CAT CGG CA 3’
PR-7	5’ TTA TTG CAA AGT ACC AGC AT 3’
vnt-pF1	5’ ATG GTA CGA GCT TGC TCC AA 3’
vnt-pR1	5’ TCA TTG GCT GCT CTC TTG AA 3’
vnt-pR2	5’ ATC AGC AGC ATC AGG TGT TG 3’
vnt-pR3	5’ GCT CTG CAA CAT CAC ACA GA 3’
PF2	5’ AGT CTT TGG GCA GAC AAC ACC AA 3’
PF2-2	5’ ATT GCA CCA TCC ACA GTC T 3’
